# Dynamics of toxigenic *Clostridium perfringens* colonisation in a cohort of prematurely born neonatal infants

**DOI:** 10.1186/s12887-020-1976-7

**Published:** 2020-02-18

**Authors:** Alexander G. Shaw, Emma Cornwell, Kathleen Sim, Hannah Thrower, Hannah Scott, Joseph C. S. Brown, Ronald A. Dixon, J. Simon Kroll

**Affiliations:** 1 Department of Infectious Disease Epidemiology, Imperial College London, London, UK; 2 Department of Medicine, Section of Paediatrics, Imperial College London, London, UK; 3 School of Life Sciences, University of Lincoln, Lincoln, UK

**Keywords:** *Clostridium perfringens*, Breast milk, Toxins, Necrotising enterocolitis

## Abstract

**Background:**

*Clostridium perfringens* forms part of the human gut microbiota and has been associated with life-threatening necrotising enterocolitis (NEC) in premature infants. Whether specific toxigenic strains are responsible is unknown, as is the extent of diversity of strains in healthy premature babies. We investigated the *C. perfringens* carrier status of premature infants in the neonatal intensive care unit, factors influence this status, and the toxic potential of the strains.

**Methods:**

*C. perfringens* was isolated by culture from faecal samples from 333 infants and their toxin gene profiles analysed by PCR. A survival analysis was used to identify factors affecting probability of carriage. Competitive growth experiments were used to explore the results of the survival analysis.

**Results:**

29.4% of infants were colonized with *C. perfringens* before they left hospital. Three factors were inversely associated with probability of carriage: increased duration of maternal milk feeds, CPAP oxygen treatment and antibiotic treatment. *C. perfringens* grew poorly in breast milk and was significantly outperformed by *Bifidobacterium infantis*, whether grown together or separately. Toxin gene screening revealed that infants carried isolates positive for collagenase, perfringolysin O, beta 2, beta, *becA/B*, *netB* and enterotoxin toxin genes, yet none were observed to be associated with the development of NEC.

**Conclusions:**

Approximately a third of preterm infants are colonised 3 weeks after birth with toxin gene-carrying *C. perfringens*. We speculate that increased maternal breast milk, oxygen and antibiotic treatment creates an environment in the gut hostile to growth of *C. perfringens*. Whilst potentially toxigenic *C. perfringens* isolates were frequent, no toxin type was associated with NEC.

**Trial registration:**

clinicaltrials.gov NCT01102738, registered 13th April 2010.

## Background

*Clostridium perfringens* is an archetypal pathosymbiont, forming part of the gut commensal microbiota in humans and animals, but also capable of producing devastating disease by way of its toxin arsenal. This anaerobic, Gram-positive spore-former is the leading cause of traumatic gas gangrene in humans [1] and one of the most common causes of food poisoning, responsible for an estimated 1 million cases in the US each year [2]. *C. perfringens* has been linked to necrotizing enterocolitis (NEC) – an inflammatory bowel disease with high mortality – in preterm neonates [3–7]. This condition has several predisposing factors; an immature gut, the presence of bacteria and potentially hypoxia and ischemia around the site of pathology [3]. A single causative organism has however remained elusive, with studies having found associations with other components of the gut microbiota such as the Enterobacteriaceae [7–9]. The toxic potential of *C. perfringens* in relation to the genesis of NEC remains intriguing. A role has also been proposed for *Clostridium* spp. in infant atopy and allergic sensitisation [10, 11] and autism [12, 13].

Previous studies investigating clostridial carriage in apparently healthy premature neonates have often involved small sample sizes, [14, 15] or reflected the opportunity to study control groups recruited for studies of topics such as the effect of different feeding or probiotic regimes [16]. We have recruited a large cohort of well-characterised premature neonates who were treated according to standard care protocols and provided faecal samples from birth until their departure from hospital. The microbiota profiles of these neonates were previously characterised by next-generation sequencing which identified significantly higher levels of *C. perfringens* in a subset of infants who were diagnosed with NEC compared to controls [7]. In the present current study, our first aim has been to use a culture-dependent approach to build up a collection of neonatal *C. perfringens* isolates and establish colonisation dynamics in the cohort. We have gone on to relate this to perinatal factors which we hypothesise will influence rates of carriage.

We have investigated the toxin gene carriage rates in these neonatal *C. perfringens* isolates. *C. perfringens* strains encode a formidable arsenal of more than 20 toxins [17, 18]- many with cytotoxic effects - including the well-characterised alpha toxin, beta toxin, and the *C. perfringens* enterotoxin (CPE, encoded by the *cpe* gene), which are respectively implicated in the human diseases gas gangrene, necrotic enteritis (Pigbel) and food poisoning (see Supplementary Material 1). Previous studies have shown that toxigenic *C. perfringens* strains are present in healthy adult populations (see Supplementary Material 1), but there is a lack of data for neonatal populations and also for toxins other than the 4 major and 2 minor toxins that make up standard multiplex PCR screens for assigning toxin type and investigating cases of food poisoning [19]. We here report the result of screening for the presence of 11 toxin genes; *cpa*, *cpb*, *etx*, *itx*, *cpe*, *cpb2*, *netB*, *becA*, *becB*, *pfoA* and *colA* (see Supplementary Material 1 for characterisations) in our neonatal isolates, seeking possible associations with NEC and establishing the normal clostridial toxin gene carriage rates in premature infants during their stay on the neonatal intensive care unit.

## Methods

### Study population

Infants born < 32 weeks gestation admitted to an Imperial College Healthcare National Health Service Trust neonatal intensive care unit (St Mary’s Hospital, Queens Charlotte’s and Chelsea Hospital) between January 2010 and December 2011 were eligible for inclusion in our ecological study “Defining the Intestinal Microbiota in Premature Infants” (The Neonatal Microbiota (NeoM) Study). Both hospitals have identical antibiotic and feeding protocols and staff members rotate between sites. Detailed daily clinical records were collected for all participants. Of the 369 babies recruited, 333 provided faecal samples for analysis in the present study (a total of 1399 samples).

### Sample collection

We collected every faecal sample produced by participants between admission and discharge. Samples were collected by nursing staff from diapers using a sterile spatula and placed in a sterile DNAase-, RNAase-free Eppendorf tube. These were stored at − 20 °C within 2 h of collection and were transferred to − 80 °C storage within 5 days. Approximately one sample per week was selected for culture.

### Clostridium culture protocol

Selective culture for clostridia species was performed using an ethanol shock methodology to eliminate non-spore forming organisms [20]. 25 mg of faeces was added to 500 μl of cooked meat broth (Oxoid) and 500 μl of 100% ethanol and vortexed for 10 sec before incubating for 30 min at room temperature. A sterile loop was dipped in the supernatant and streaked onto a fastidious anaerobic agar plate supplemented with 0.1% sodium taurocholate hydrate (Sigma) and 5% defibrinated sheep blood (Oxoid). Plates were incubated anaerobically at 37 °C for 48 h in an Oxoid 2.5 l AnaeroJar plus AnaeroGen sachet. Resulting colonies were examined and for each morphologically distinct isolate, four colonies were subcultured by streaking onto Colombia agar with sheep blood (Oxoid) plates which were incubated anaerobically at 37 °C for 48 h. Single colonies were used for identification to the species level by matrix-assisted laser desorption/ ionization–time of flight (MALDI-TOF) using a Bruker Microflex LT (Bruker Daltonics), and the remainder of the subculture stored in 70% brain heart infusion (BHI) broth (Oxoid), 30% glycerol (Sigma) at − 80 °C.

### Toxin typing of *Clostridium perfringens* by polymerase chain reaction (PCR)

PCR reactions were performed to detect *cpa*, *cpb*, *iA*, *etx*, *cpe* and *cpb2* (multiplex PCR), *becA* and *becB* (duplex PCR) and *netB, pfoA*, and *colA* (single PCR) and visualised by gel electrophoresis. For details see Supplementary Material 2.

### Breast milk growth competition experiment

*Bifidobacterium infantis* (NCIMB 702255 – isolated from infant intestine), and *Clostridium perfringens* isolated from the faeces of an infant enrolled in our study were cultured either in Wilkins-Chalgren broth (WCB) (Oxoid) supplemented with 5 g/l soya peptone (Oxoid), or human breast milk. Three donors gave breast milk on three occasions, for which informed consent was obtained and standardised expression protocols used. Breast milk was expressed on average 16 h before each experiment, was kept refrigerated at all times, and had a commensal microbial load (anaerobic) below the NICE-recommended limit (10^5^ colony forming units (CFU)/ml total viable organisms or 10^4^ CFU/ml Enterobacteriaceae or *Staphylococcus aureus*) [21], which was calculated by plating directly onto Colombia agar with sheep blood (Oxoid) and incubating for 48 h at 37 °C, followed by species identification using MALDI-TOF. The majority of organisms found in this manner were *Staphylococcus* and *Actinomyces* spps. *Staphylococcus* spp. were grown in every breast milk sample, whilst *Actinomyces* spp. were found in the milk of only one mother (see Supplementary Material 3). For bacterial counts, *C. perfringens* was grown on Tryptose Sulphite Cycloserine (TSC) agar plates (Oxoid) [22], and incubated for 24 h at 37 °C, and *B. infantis* on Bifidus Selective Medium (BSM) agar plates (Sigma) incubated for 48 h at 37 °C. All cultivation was performed under anaerobic conditions using an Oxoid 2.5 l AnaeroJar plus AnaeroGen sachet in a shaking or static 37 °C incubator.

Bacteria were grown in WCB to an optical density of approximately 0.7, and approximately 1 × 10^4^ colony forming units (CFUs) added to 2.5 ml of breast milk or 2.5 ml of WCB in 15 ml bioreactor tubes (TTP). Media were inoculated with *C. perfringens* or *B. infantis* (monoculture), or both (co-culture). *C. perfringens* and *B. infantis* were enumerated after 20 h incubation by plating dilutions in triplicate onto TSC agar and BSM agar respectively. Breast milk from three different donors was used and experiments carried out in triplicate (to total nine experiments).

### Statistics

Statistical analyses were performed in the R statistical package (version 3.3.1). A Cox Proportional-Hazard regression function from the ‘survival’ package was used for the survival analysis. An initial univariate analysis was performed to determine any significant relation between each clinical factor in Table 1 and *C. perfringens* colonisation (defined as the earliest faecal sample where *C. perfringens* was isolated). Factors found to remain significant after a multiple hypothesis (Bonferroni) correction were entered into an iterative multivariate model, with factors found to have a *p* value of > 0.1 being removed at each iterative step. Barnard’s test was performed to detect associations between antibiotic use and breast milk feeds and long-term carriage, and between toxin genes and NEC development. Growth rate comparisons were performed using generalized linear models and the ‘mass’ package using both the mean fold-change for each experimental triplicate and the mean absolute change in CFU. *P*-values shown were derived from the fold-change models, with similar results by either metric.

**Table 1:** Demographics of infants included in the analysis (*N* = 333)

Demographics	Number
Gestation	
Mean gestation at birth in days (SD)	197 (16)
Median gestation at birth in days (IQR)	198 (27)
Birth weight	
Mean birth weight in g (SD)	1079.5 (340.8)
Median birth weight in g (IQR)	1025 (525)
Gender	
Female (%)	155 (46.5%)
Male (%)	178 (54.5%)
Ethnicity	
White (%)	130 (39.0%)
Mixed (%)	44 (13.2%)
Asian (%)	58 (17.4%)
Black (%)	74 (22.2%)
Unknown (%)	12 (3.6%)
Other (%)	15 (4.5%)
Mode of delivery	
Vaginal delivery (%)	137 (41.1%)
C-section (%)	196 (58.9%)
Ventilation	
Mean number of days requiring mechanical ventilation prior to CPC or LS (SD)	3.1 (8.2)
Median number of days requiring mechanical ventilation prior to CPC or LS (IQR)	1 (2)
Mean number of days CPAP (air) prior to CPC or LS (SD)	7.0 (8.7)
Median number of days CPAP (air) prior to CPC or LS (IQR)	4 (10)
Mean number of days CPAP (oxygen) prior to CPC or LS (SD)	9.3 (14.8)
Median number of days CPAP (oxygen) prior to CPC or LS (IQR)	2 (12)
Feeding	
Mean number of days of donor breast milk prior to CPC or LS (SD)	8.3 (8.9)
Median number of days of donor breast milk prior to CPC or LS (IQR)	6 (8)
Mean number of days of maternal breast milk prior to CPC or LS (SD)	23.0 (22.2)
Median number of days of maternal breast milk prior to CPC or LS (IQR)	17 (25)
Mean number of days of formula prior to CPC or LS (SD)	3.3 (9.0)
Median number of days of formula prior to CPC or LS (IQR)	0 (2)
Mean number of days of breast feeding prior to CPC or LS (SD)	3.4 (7.4)
Median number of days of breast feeding prior to CPC or LS (IQR)	0 (3)
Antibiotic use	
Mean number of days of antibiotic use at birth (SD)	2.4 (2.1)
Median number of days of antibiotic use at birth (IQR)	2 (2)
Mean number of days of antibiotic use prior to CPC or LS (SD)	5.1 (7.4)
Median number of days of antibiotic use prior to CPC or LS (IQR)	3 (4)
*C. perfringens* colonisation	
Number colonised with *C. perfringens* (%)	98 (29.4%)
Mean number of days prior to CPC (SD)	27.1 (23.3)
Median number of days prior to CPC (IQR)	21 (26)

Abbreviations: *CPC* C*. perfringens* colonisation, *LS* Last sample, *CPAP*, Continuous positive airway pressure, *SD* Standard deviation, *IQR* Interquartile range

## Results

### *C. perfringens* incidence and clinical factors associated with its colonisation

Faecal samples and complete clinical notes were available for 333 infants. The demographics of these babies are shown in Table 1. *C. perfringens* was isolated in faecal samples from 98 of the infants (29.4%).

**Fig. 1:**
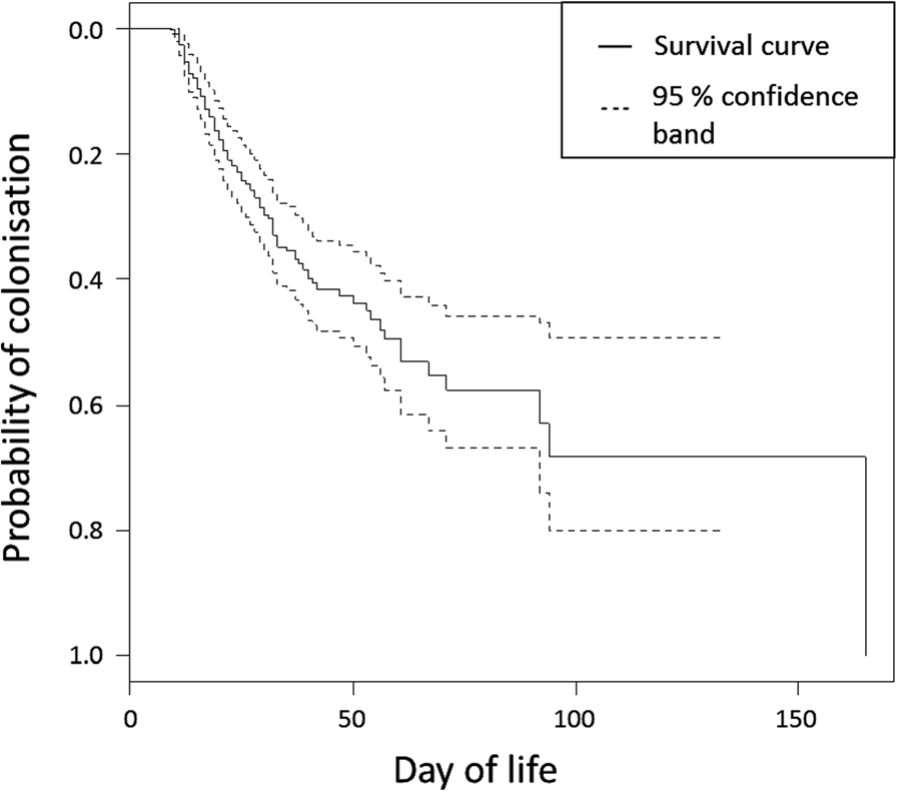
Kaplan-Meier plot of probability of colonisation by *C.* *perfringens* over time. Data from our cohort of 333 infants. Dashed lines indicate the Hall-Wellner 95% confidence bands

Colonisation data were used to predict the risk of colonisation of the infant gut by *C. perfringens* over time through a survival analysis (Fig. 1). By the day of life of the median stay on the neonatal unit for the cohort (29 days), a predicted 36% of infants would be colonised (95% confidence band 25, 43%).

We next repeated the survival analysis with the aim of determining clinical variables (shown in Table 1) that were associated with *C. perfringens* colonisation. A series of univariate models were created and significantly associated variables (after multiple hypothesis correction) are shown in Table 2.

**Table 2:** Results of the univariate survival analysis

Variable	Quartiles					Corrected *P* value	Coefficient	Exponentiated Coefficient (95% CI)	Risk change for 75% Quartile
	0%	25%	50%	75%	100%				
Gestation (days)	161	184	198	211	223	0.002	0.026	1.026 (1.013–1.040)	3.688
Birth weight (g)	500	800	1025	1325	1890	< 0.001	0.001	1.001 (1.001–1.002)	2.788
Days of mechanical ventilation	0	0	1	2	84	0.021	−0.076	0.927 (0.885–0.971)	0.860
Days of CPAP oxygen	0	0	2	12	80	< 0.001	−0.039	0.962 (0.947–0.977)	0.626
Days of maternal milk feeds (non-breast)	0	6	14	29	114	< 0.001	−0.057	0.945 (0.930–0.959)	0.192
Days of breast feeds	0	0	0	3	48	0.001	−0.073	0.929 (0.896–0.964)	0.802
Days of antibiotic use	0	2	3	6	79	< 0.001	−0.116	0.89 (0.848–0.934)	0.497

For each significant factor, values are provided at each of its quartiles to illustrate the spread of data. The exponentiated coefficients provide the change in risk of colonisation per unit of each factor. Risk change indicates the relative change in risk of colonisation between the minimum value (0%) and the 75% quartile for a given clinical factor. Abbreviations: CI, confidence interval.

**Fig. 2:**
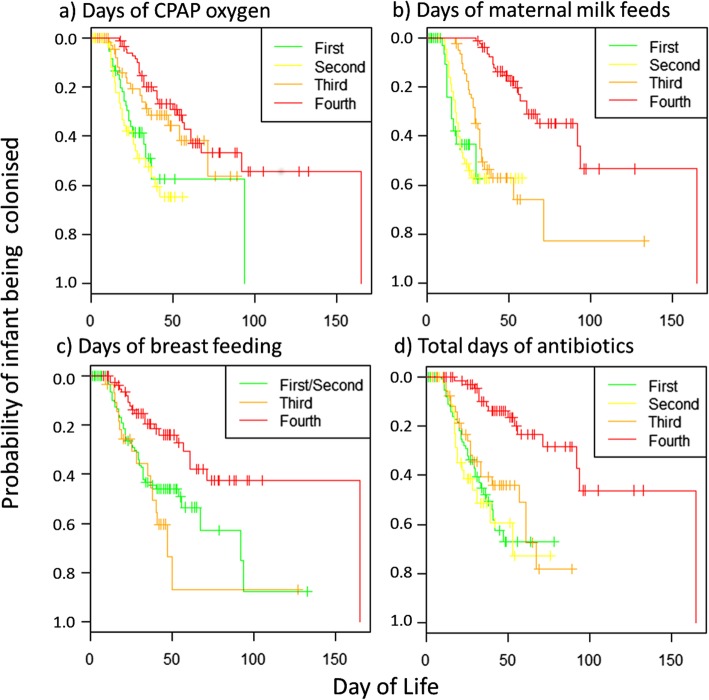
Kaplan-Meier plots for each of the four significant factors in the multivariate model**.** X axis shows the infant day of life. Y axis shows the probability of colonisation for an infant when stratified according to quartiles (1st – 4th) of varying clinical factors: **a**) Days of CPAP oxygen, **b**) Days of maternal milk feeds (excluding breast feeds), **c**) Days of breast feeding and d) Days of antibiotics usage. Colour codes for the quartiles are shown in the top right of each subplot

Given the potential for correlation between these variables, a multivariate survival analysis was used to identify a minimal set of clinical factors to best predict colonisation. Four factors were found to remain significant in this model, with associations between increased probability of *C. perfringens* colonisation and fewer days of CPAP with supplemental oxygen (CPAP oxygen), fewer days of maternal milk feeds (via feeding tube), fewer days of breast feeding and fewer days of antibiotics over the course of the infant’s admission. The variation of colonisation probabilities when the infant cohort is divided into quartiles for each of these factors is shown in Fig. 2.

**Fig. 3:**
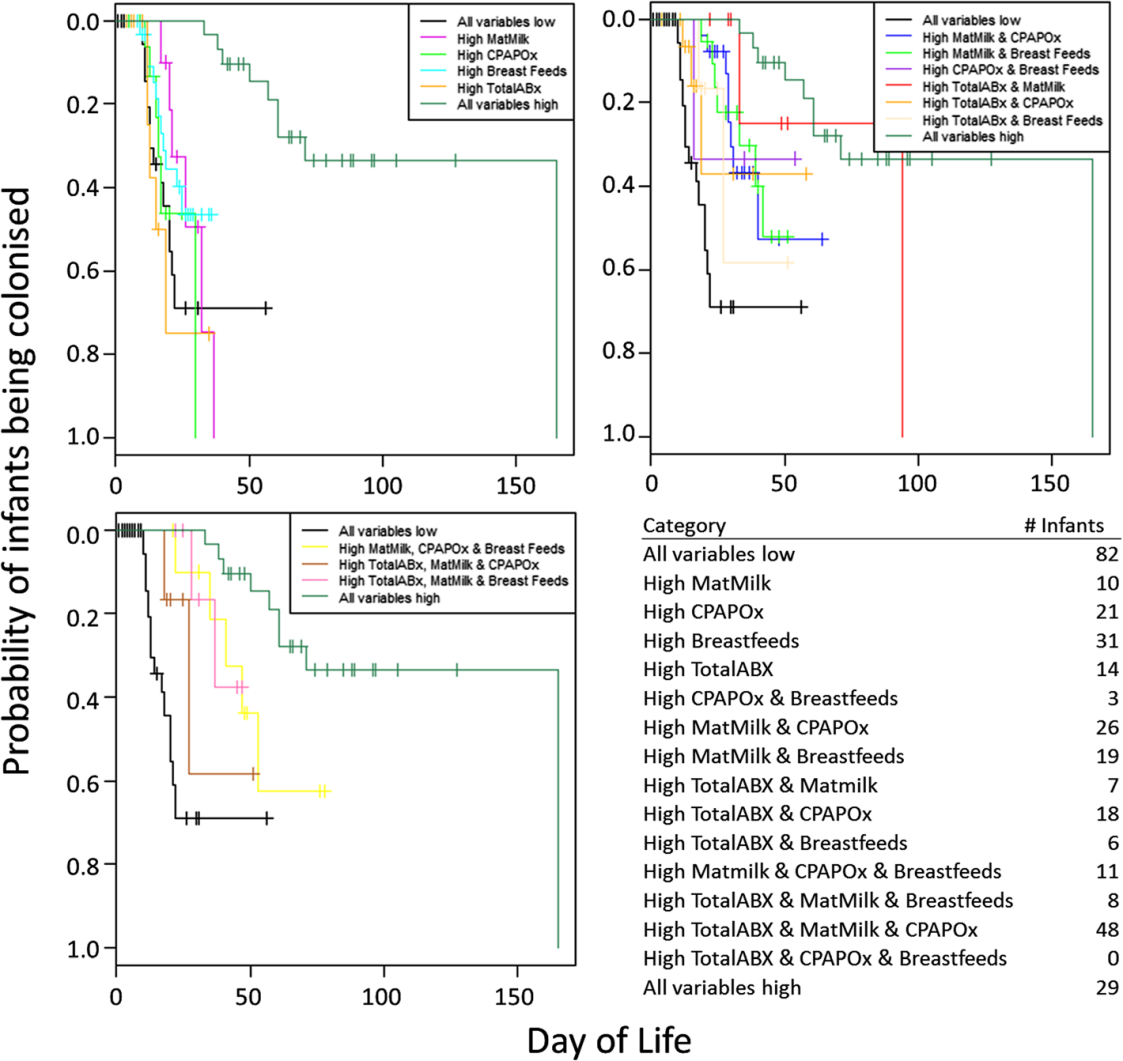
Kaplan-Meier plots showing combinations of the four significant factors when split into low or high categories. For ease of comparability, ‘All variables low’ (<= median value for all four variables) and ‘All variables high’ (>median value for all four variables) are shown on each chart

As the risk of colonisation for each infant is associated with the combined effects of each of these factors, we performed a multivariate analysis where each infant in the cohort was annotated either “low” (<= median) or “high” (>median) for each of the four factors. Multiple sets of analyses were run, illustrating the effects of higher than average measure of a single factor, or combinations thereof, on the probability of colonisation. The results are shown in Fig. 3.

The dominant variable of the four appeared to be feeds with breast milk, with increased feeds being associated with the greatest shift towards low probability of *C. perfringens* colonisation. We theorised two modes of action for this association, with breast milk acting either directly (inhibiting the growth of *C. perfringens*) or indirectly (promoting the growth of other components of the gastro-intestinal microbiota which outcompete *C. perfringens*). We explored these possibilities in culture experiments as follows.

### Growth of *C. perfringens* in breast milk

**Fig. 4:**
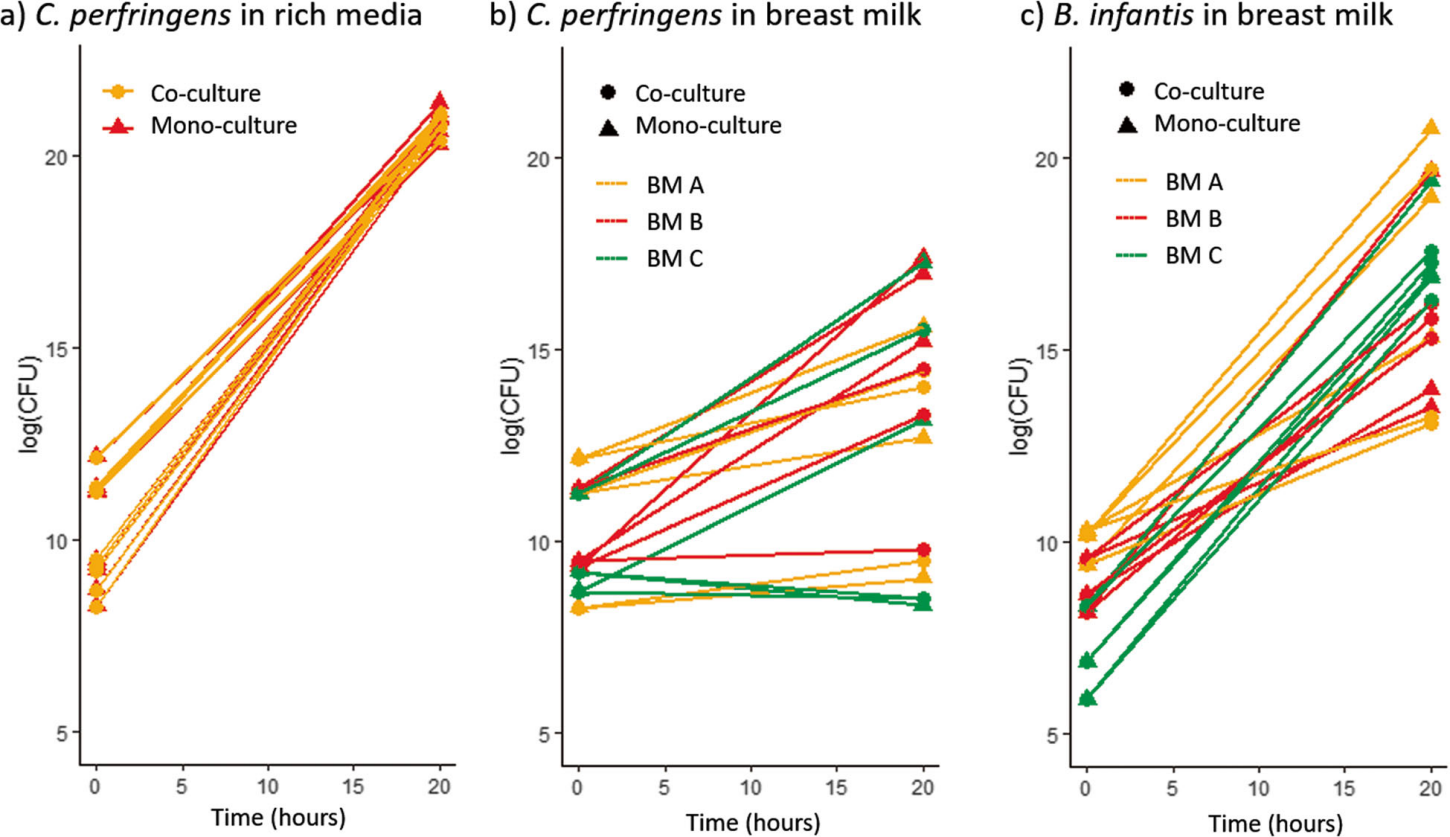
*C. perfringens* and *B. infantis* growth in rich media and breast milk. Each bacterium was grown in each medium separately (monoculture) or together (co-culture). Experiments were performed in triplicate with three technical replicates (all replicas shown). BM = Breast milk, with three different donations being used in the experiments (**a**, **b** and **c**)

A *C. perfringens* isolate was grown in either nutrient rich medium (supplemented WCB broth) or breast milk, either as a monoculture or in co-culture with *Bifidobacterium infantis* which was chosen to represent a typical competing gut species, one that thrives on breast milk oligosaccharides [23–25]. Both species grew in each substrate and under both culture conditions, and each grew significantly better in rich medium under monoculture than in breast milk (*B. infantis*, *p* = 0.008, *C. perfringens*, *p* < 0.0001). There was no significant difference in growth between the two species in the rich media when comparing the fold change between inoculation and the 20-h timepoint. However, in breast milk *C. perfringens* grew at a significantly lower rate than *B. infantis* in monoculture (*p* = 0.003) and co-culture (*p* = 0.0001). *B. Infantis* growth in breast milk was unaffected by mono- or co-culture, whilst growth of *C. perfringens* varied greatly but without significant association to breast milk type or mono- or co-culture (Fig. 4).

### Longitudinal carriage of *C. perfringens*

We sought to establish whether mother’s milk feeds or antibiotic treatments after the initial colonisation affected carriage of *C. perfringens*. 68 infants had at least one additional sample available after *C. perfringens* was first identified. Of these infants, 28% (*n* = 19) yielded no further isolates. For 31% of infants (*n* = 21), all subsequent samples were positive, while the remaining 41% of infants (*n* = 28) tested positive in at least half of their additional samples. We recorded whether the infants recevied maternal milk feeds, CPAP oxygen or antibiotic treatment in the week after their initial colonisation, and found none of the factors to be significantly asscoated with multiple isolation events compared to a single isolation (*p* = 0.75, 0.88 and 0.80 respectively).

### Toxic potential of *C. perfringens* isolates

**Fig. 5:**
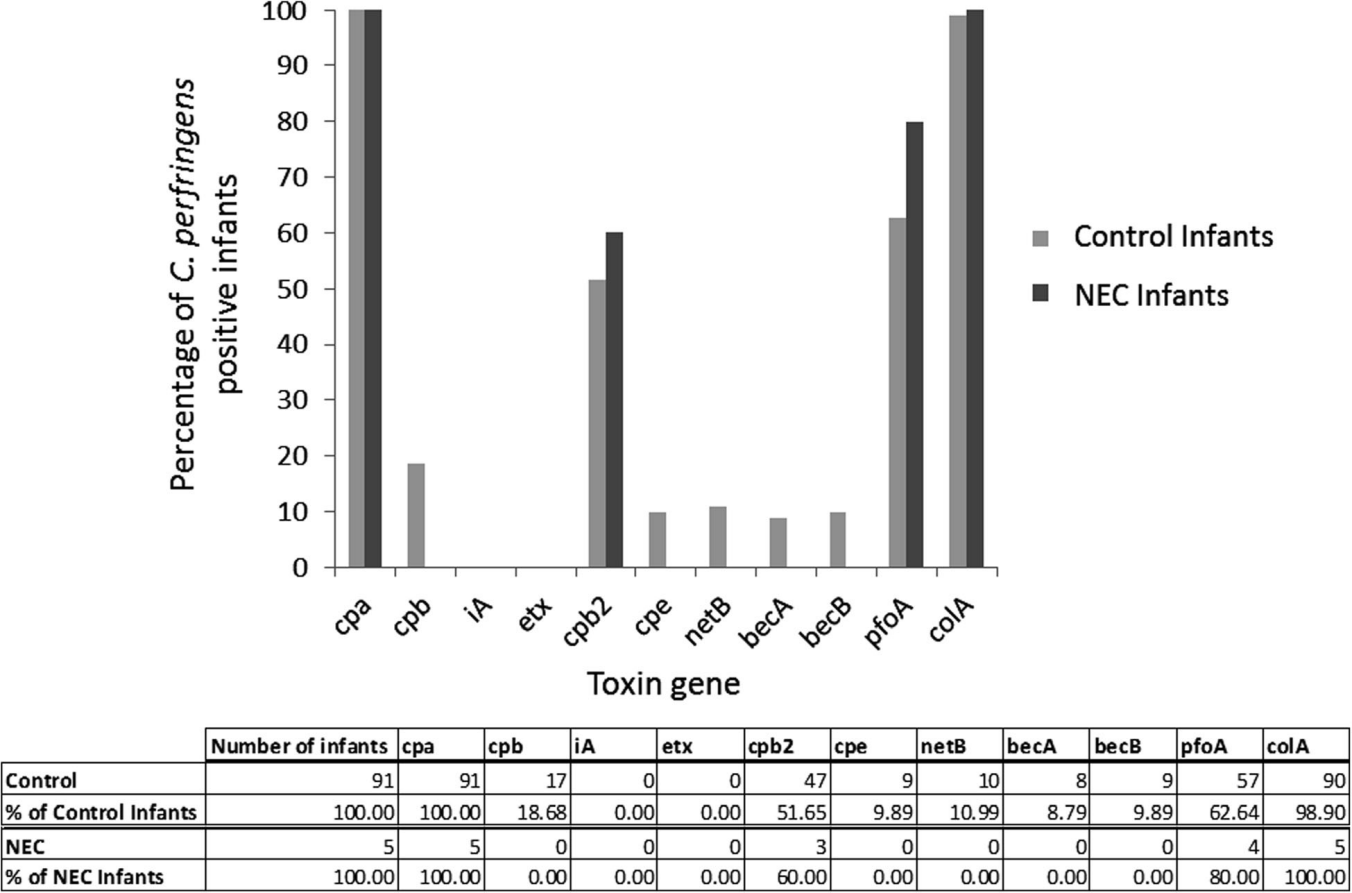
*C. perfringens* toxin genes found in infants that developed NEC compared to control infants. Percentages were calculated out of the total number of control infants (*n* = 91) and infants that developed NEC Bell stage 2 (confirmed) or 3 (severe) (*n* = 5). Two infants who developed NEC Bell stage 1 (suspected) could not be categorised as cases or controls so were not included. A toxin gene was scored as present if it was found in any *C. perfringens* isolate found in an infant’s faecal samples during the course of either their whole time on the neonatal unit or up to the last sample prior to NEC development. The table shows the counts within the two groups and the relative percentages for toxin occurrence

The harmful effects of *C. perfringens* arise in large part through the production of toxins. Of particular interest in the neonatal field is the potential for *C. perfringens* toxin to play a part in the pathogenesis of NEC [4, 5]. We surveyed the *C. perfringens* isolates derived from our cohort for the presence of toxin genes through targeted PCR and found that a range of toxin genes were present. The presence of toxin genes in *C. perfringens* isolates during any point in their admission was scored for each neonate, allowing comparison of the toxic potential of the isolates prior to either discharge from the neonatal intensive care unit (“Control infants”) or NEC incidence (“NEC Infants”) (see Fig. 5).

Statistical analyses (Barnard’s test and survival analysis) found no significant associations between the presence of toxin genes and the development of NEC. Given the infant numbers available in our cohort, Barnard’s test would detect a proportional increase of 0.19 or greater in the occurrence of toxin in infants developing NEC compared to controls with 95% confidence (assuming a one-sided test and 80% power). These results therefore do not support the hypothesis of NEC being associated with the prevalence of a particular toxin gene across our infant cohort. Overall, the most prevalent toxin genes in our neonatal *C. perfringens* isolates were *cpa*, *pfoA*, *colA* and *cpb2*, which were found in 100, 64, 99 and 50% of isolates respectively.

## Discussion

We have found that just under a third of infants (29.4%) in our premature neonatal cohort were colonised with *C. perfringens* in their gut at some time during their stay in the neonatal intensive care unit (NICU), with duration of maternal milk feeds, antibiotic therapy, and continuous positive airway pressure with supplemental oxygen (CPAP oxygen) treatment exerting the strongest influence over probability of carriage. Our reported risk of colonisation with *C. perfringens* over time (approximately 25% at 3 weeks and 44% at 3 weeks) is very similar to findings in some other culture-dependent studies (35% at 3 weeks of life [14], 46.1% at 7 weeks of life [16]), although we are aware of one report of higher rates early in life (56.5% at 1 week of life [26]).

We have found a significant inverse association between both duration of maternal milk feeds and breast feeding and probability of *C. perfringens* colonisation. This is in line with studies of term infants, which have reported that formula-fed infants have higher gut Clostridia counts compared to those who were breast-fed [27–29]. Bioactive constituents (immunoglobulins, lysozyme, lactoferrin, antimicrobial peptides, oligosaccharides) and commensal microbes in breast milk [30] actively protect against pathogen colonisation and invasion in the neonatal gut. An important mechanism of action is efficient metabolism of human milk oligosaccharides (HMOs) and subsequent acid production, for which *B. infantis* is especially adapted [23]. Many other gut commensal species, including *Clostridium* species [24, 25, 31], cannot metabolise HMOs. Our growth experiments have shown that *B. infantis* significantly outperforms *C. perfringens* when grown in monoculture (*p* = 0.003) and co-culture (*p* = 0.0001) in breast milk. When the two species were cultured together, *C. perfringens* counts were not significantly reduced in the presence of *B. infantis* (*p* = 0.1083) hence we saw no evidence of direct inhibitory action. This comparison could be further confounded by a range of other factors; potential competition by low abundance organisms derived from the breast milk (predominantly Staphylococci), the impact of *C. perfringen* toxins on the growth of Bifidobacteria and the effect of potential oxygen exposure which would impact *C. perfringens* more strongly than *Bifidobacterium* [32]. Direct inhibition by specific *Bifidobacterium* species has however been previously reported for growth of enteric pathogens (including *C. perfringens*) [33, 34] in vitro; for growth of NEC-associated clostridia in a quail model [35]; and for clostridial growth in mouse models [36]. It has recently been reported that term infants who are carriers of *C. perfringens* have consistently lower levels of Bifidobacteria in their gut compared to non-carriers [37].

Interestingly, the impact of breast milk on *C. perfringens* carriage we report was only found for infants fed their own mother’s milk (breast-fed or expressed into bottles), and not for those fed donor milk. In a recent systematic review [38], heat-treating donor milk to meet safety standards (normally by Holder pasteurization - 62.5 °C for 30 min, [21]), was shown consistently to reduce the level and/or activity of milk proteins including immunoglobulins, lactoferrin and enzymes, but had no effect on the lipid or saccharide content of milk. This suggests that donor milk may be inferior to maternal milk in an immunological capacity only, retaining qualities sufficient to influence *C. perfringens* colonisation patterns in some contexts. It has recently been demonstrated that neonatal mice can be protected from enteric infection by antibodies delivered through breast milk [39]. The absence of competing milk commensals in donor milk may perhaps further explain why in our study it failed to influence *C. perfringens* carriage in the way that maternal milk did.

Our results indicate that prolonged treatment with antibiotics or CPAP oxygen creates a hostile environment for *C. perfringens* in the preterm gut, reducing probability of carriage. *C. perfringens* may be particularly impacted by antibiotics due a rarity of multiple drug resistance genes [40] compared to other members of the neonatal gut microbiota [41]. A reduction in the abundance of *C. perfringens* in the preterm infant gut with antibiotic exposure has been observed in another cohort, although not reduced prevalence [16]. While antibiotic prophylaxis is routine in preterm neonatal care, it has been shown to significantly alter the gut microbiota in preterm infants [42, 43] and to be associated with adverse health outcomes including NEC, sepsis and death [44, 45]; the benefits of avoiding putative *C. perfringens*-induced pathologies such as NEC must be weighed against the risks of inducing gut dysbiosis when considering antibiotic regimes.

Similar to Ferraris et al., [16] we found that delivery mode had no effect on *C. perfringens* colonisation rates in our preterm neonatal cohort, in contrast to term infants [46–48].

Our analysis of the longitudinal carriage of *C. perfringens* found no association between maternal milk feeds, CPAP oxygen or antibiotic treatment in the week after the initial isolation *of C. perfringens* and continued carriage (defined as at least half of subsequent samples testing positive). Our study may however be underpowered to conclude whether these factors truly influence long term carriage as there was limited time for observation between colonisation and the infants being discharged from the NICU.

Our toxin typing results in a limited number of *C. perfringens*-associated NEC cases did not identify a specific toxin-mediated pathology. They have however provided evidence of a high occurrence of multi-toxigenic *C. perfringens* strains in the premature neonatal gut. The three most prevalent toxin genes in our neonatal *C. perfringens* isolates (*cpa*, *pfoA* and *colA*) can all be located to the same extracellular toxin gene cluster within a 250 kb region on the chromosome [49]. Of note was the presence of the plasmid-encoded *beta2* toxin gene, which was found in50% of *C. perfringens*-positive infants. Although not fully elucidated, this toxin is strongly associated with porcine necrotic enteritis and is cytotoxic for human colorectal epithelial (CaCo-2) cells [50]. Carriage of toxin genes, including *beta2,* is however observed in healthy human populations [51], hence the toxic phenotype is likely highly situational.

We detected other plasmid-encoded toxin genes in our neonatal isolates that have previously almost exclusively been detected in *C. perfringens* isolates from non-human species or cases of specific diseases: for example, the *netB* gene and necrotic enteritis in poultry, and *becAB* genes and food poisoning in Japan. The most notable example of this however, is the presence of the beta toxin gene (denoting type C *C. perfringens* when found with the alpha toxin and no other major toxin) in 17 infants. Type C *C. perfringens* in humans is normally only isolated in cases of necrotic enteritis (Pigbel) or from healthy humans in endemic areas. We hypothesise that there is a low level circulation of “aberrant” toxin genes in the neonatal population, presumably acquired from the mother at birth, and detectable in this study due to large sample sizes (273 isolates screened). It is important to consider however, the high level of discordance between genotype and phenotype for many of the *C. perfringens* toxins.

## Conclusions

We sought to understand the *C. perfringens* colonisation dynamics in a cohort of infants at high risk of mortality and morbidity from clostridia GI-induced pathologies. We used a traditional culture method combined with PCR to demonstrate that toxigenic *C. perfringens* is part of the normal gut microbiota in preterm neonates and report that approximately 30% are colonised before they leave the NICU. We have identified perinatal factors that are able to significantly affect the probability of *C. perfringens* carriage: increased duration of maternal milk feeds, increased duration of CPAP oxygen treatment, and increased duration of antibiotic treatment, which correlated with protection *from C. perfringens* colonisation. We demonstrated an inhibitory effect of breast milk on the growth of *C. perfringens* in vitro and reaffirm the importance of maternal milk feeding in preterm neonatal care.

## Supplementary information

**Additional file 1. ***C. perfringens* toxin genes screened for in this study.

**Additional file 2.** PCR methods for toxin gene detection.

**Additional file 3.** Organisms grown in breast milk screening

## Data Availability

Not applicable.

## Abbreviations

BHI: Brain heart infusion
BSM: Bifidus selective medium
CFU: Colony forming units
CI: Confidence interval
CPAP: Continuous positive airway pressure
CPC: *C. perfringens* colonisation
CPE: *C. perfringens* enterotoxin
HMO: Human milk oligosaccharides
IQR: Interquartile range
LS: Last sample
MALDI-TOF: Matrix-assisted laser desorption/ ionization–time of flight
NEC: Necrotising enterocolitis
NICU: Neonatal intensive care unit
PCR: Polymerase chain reaction
SD: Standard deviation
TSC: Tryptose sulphite cycloserine
WCB: Wilkins-chalgren broth
