# Supplementary material for: Dynamics of toxigenic Clostridium perfringens colonisation in a cohort of prematurely born neonatal infants
Source: BMC Pediatr. 2020 Feb 18;20:75. doi: 10.1186/s12887-020-1976-7 (PMC7027286; doi:10.1186/s12887-020-1976-7)
Supplement: Supplementary file 1 — Additional file 1. C. perfringens toxin genes screened for in this study. [file 12887_2020_1976_MOESM1_ESM.docx]

**Supplementary Material** **1**

***C. perfringens* toxin genes screened for in this study (N=11).**

| Toxin, gene | Toxin type  Mode of action | Role in human disease | Prevalence in health | Cohort | Reference |
| --- | --- | --- | --- | --- | --- |
| Alpha, cpa | Major, toxin type A  Phospholipase C [1] | Gas gangrene [2] | ^a^33-63% | Adults | [3, 4] |
|  |  |  | ^a^35-56.5% | Preterm neonates | [5-7] |
| Beta, cpb | Major, toxin type C  Pore-forming toxin (PFT) [8] | Necrotic enteritis (Pigbel/ Darmbrand) [9-11] | 0% | All (adults, neonates etc) | [12] |
|  |  |  | 75% | Pigbel endemic regions eg: Papua New Guinea | [12] |
| Epsilon, etx | Major, toxin type B (when with beta) and toxin type D  PFT [13] | Not reported  (^b^Type B and D cause necrohemorrhagic enteritis and enterotoxemia in animals respectively [14]) | NA | NA | NA |
| Iota, itx | Major, toxin type E  Actin-specific ADP-ribosyltransferase [15] | Not reported  (^b^Putative role in enteritis in cattle, sheep & rabbits [14]) | NA | NA | NA |
| Enterotoxin, cpe | Minor  PFT [16] | Food poisoning, [17] sporadic diarrhoea (SD) & antibiotic-associated diarrhoea (ADD) [18] | 1-5% | Global isolates | [19] |
|  |  |  | 7-18% | Adults | [4, 20] |
|  |  |  | 1.1%, 4.5%, 10.1% | 1, 3, 6-month term infants | [21] |
| Beta 2, cbp2 | Minor  Putative PFT [14] | Inconclusive, putative role in enteric disease [22] | 30.2% | Adults | [4] |
|  |  |  | 38% | Children | [23] |
| Necrotic enteritis-like Toxin B, netB | Minor  PFT [24] | Not reported  (^b^Virulence factor in necrotic enteritis in poultry [24]) | 7.5% | Children | [25] |
| Binary enterotoxin of *C. perfringens*, becA & becB | Minor  ADP-ribosylating binary toxin [26] | Food poisoning [26] | < 1% | Adults | [27] |
| Theta (perfringolysin O), pfoA | Minor  PFT (cholesterol-dependent cytolysin) [28] | Thought to work synergistically with cpa in gas gangrene progression [29, 30] | 81.8% | *C. perfringens* isolates from healthy adults (N=11) | [31] |
| Kappa (collagenase), colA | Minor, extracellular collagenase [32] | Not reported | 100% | *C. perfringens* isolates from healthy adults (N=11) | [31] |

*C. perfringens* toxin genes screened for in this study, their mode of action, role in human disease and prevalence in *C. perfringens* isolated from healthy populations.

^a^ *cpa* is carried by all *C. perfringens* strains and toxinotypes; prevalence of this toxin is therefore the same as the *C. perfringens* carriage levels in a population

^b^ *C. perfringens* toxin types involved in diseases of animals

**References**

1. Titball RW, Naylor CE, Basak AK: **The Clostridium perfringens alpha-toxin**. *Anaerobe* 1999, **5**(2):51-64.

2. Awad MM, Bryant AE, Stevens DL, Rood JI: **Virulence studies on chromosomal alpha-toxin and theta-toxin mutants constructed by allelic exchange provide genetic evidence for the essential role of alpha-toxin in Clostridium perfringens-mediated gas gangrene**. *Molecular microbiology* 1995, **15**(2):191-202.

3. Nagpal R, Ogata K, Tsuji H, Matsuda K, Takahashi T, Nomoto K, Suzuki Y, Kawashima K, Nagata S, Yamashiro Y: **Sensitive quantification of Clostridium perfringens in human feces by quantitative real-time PCR targeting alpha-toxin and enterotoxin genes**. *BMC microbiology* 2015, **15**:219.

4. Carman RJ, Sayeed S, Li J, Genheimer CW, Hiltonsmith MF, Wilkins TD, McClane BA: **Clostridium perfringens toxin genotypes in the feces of healthy North Americans**. *Anaerobe* 2008, **14**(2):102-108.

5. Blakey JL, Lubitz L, Barnes GL, Bishop RF, Campbell NT, Gillam GL: **Development of gut colonisation in pre-term neonates**. *Journal of medical microbiology* 1982, **15**(4):519-529.

6. Ferraris L, Butel MJ, Campeotto F, Vodovar M, Rozé JC, Aires J: **Clostridia in Premature Neonates' Gut: Incidence, Antibiotic Susceptibility, and Perinatal Determinants Influencing Colonization**. *PLoS ONE* 2012, **7**(1):e30594.

7. Rotimi VO, Olowe SA, Ahmed I: **The development of bacterial flora of premature neonates**. *The Journal of Hygiene* 1985, **94**(3):309-318.

8. Steinthorsdottir V, Halldorsson H, Andresson OS: **Clostridium perfringens beta-toxin forms multimeric transmembrane pores in human endothelial cells**. *Microbial pathogenesis* 2000, **28**(1):45-50.

9. Walker PD, Foster WH, Knight PA, Freestone DS, Lawrence G: **Development, preparation and safety testing of a Clostridium welchii type C toxoid. I: Preliminary observations in man in Papua New Guinea**. *Journal of Biological Standardization* 1979, **7**(4):315-323.

10. Fisher DJ, Fernandez-Miyakawa ME, Sayeed S, Poon R, Adams V, Rood JI, Uzal FA, McClane BA: **Dissecting the contributions of Clostridium perfringens type C toxins to lethality in the mouse intravenous injection model**. *Infection and immunity* 2006, **74**(9):5200-5210.

11. Sayeed S, Uzal FA, Fisher DJ, Saputo J, Vidal JE, Chen Y, Gupta P, Rood JI, McClane BA: **Beta toxin is essential for the intestinal virulence of Clostridium perfringens type C disease isolate CN3685 in a rabbit ileal loop model**. *Molecular microbiology* 2008, **67**(1):15-30.

12. Lawrence G, Walker PD: **Pathogenesis of enteritis necroticans in Papula New Guinea**. *Lancet* 1976, **1**(7951):125-126.

13. Miyata S, Matsushita O, Minami J, Katayama S, Shimamoto S, Okabe A: **Cleavage of a C-terminal peptide is essential for heptamerization of Clostridium perfringens epsilon-toxin in the synaptosomal membrane**. *The Journal of biological chemistry* 2001, **276**(17):13778-13783.

14. Uzal FA, Vidal JE, McClane BA, Gurjar AA: **Clostridium Perfringens Toxins Involved in Mammalian Veterinary Diseases**. *The open toxinology journal* 2010, **2**:24-42.

15. Vandekerckhove J, Schering B, Barmann M, Aktories K: **Clostridium perfringens iota toxin ADP-ribosylates skeletal muscle actin in Arg-177**. *FEBS letters* 1987, **225**(1-2):48-52.

16. Kitadokoro K, Nishimura K, Kamitani S, Fukui-Miyazaki A, Toshima H, Abe H, Kamata Y, Sugita-Konishi Y, Yamamoto S, Karatani H *et al*: **Crystal Structure of Clostridium perfringens Enterotoxin Displays Features of β-Pore-forming Toxins**. *The Journal of biological chemistry* 2011, **286**(22):19549-19555.

17. Sarker MR, Carman RJ, McClane BA: **Inactivation of the gene (cpe) encoding Clostridium perfringens enterotoxin eliminates the ability of two cpe-positive C. perfringens type A human gastrointestinal disease isolates to affect rabbit ileal loops**. *Molecular microbiology* 1999, **33**(5):946-958.

18. Collie RE, McClane BA: **Evidence that the enterotoxin gene can be episomal in Clostridium perfringens isolates associated with non-food-borne human gastrointestinal diseases**. *Journal of Clinical Microbiology* 1998, **36**(1):30-36.

19. McClane BA: **An overview of Clostridium perfringens enterotoxin**. *Toxicon : official journal of the International Society on Toxinology* 1996, **34**(11-12):1335-1343.

20. Heikinheimo A, Lindstrom M, Granum PE, Korkeala H: **Humans as reservoir for enterotoxin gene--carrying Clostridium perfringens type A**. *Emerging infectious diseases* 2006, **12**(11):1724-1729.

21. Nagpal R, Tsuji H, Takahashi T, Nomoto K, Kawashima K, Nagata S, Yamashiro Y: **Gut dysbiosis following C-section instigates higher colonisation of toxigenic Clostridium perfringens in infants**. *Beneficial microbes* 2017, **8**(3):353-365.

22. Fisher DJ, Miyamoto K, Harrison B, Akimoto S, Sarker MR, McClane BA: **Association of beta2 toxin production with Clostridium perfringens type A human gastrointestinal disease isolates carrying a plasmid enterotoxin gene**. *Molecular microbiology* 2005, **56**(3):747-762.

23. Finegold SM, Summanen PH, Downes J, Corbett K, Komoriya T: **Detection of Clostridium perfringens toxin genes in the gut microbiota of autistic children**. *Anaerobe* 2017, **45**:133-137.

24. Keyburn AL, Boyce JD, Vaz P, Bannam TL, Ford ME, Parker D, Di Rubbo A, Rood JI, Moore RJ: **NetB, a New Toxin That Is Associated with Avian Necrotic Enteritis Caused by Clostridium perfringens**. *PLoS Pathogens* 2008, **4**(2):e26.

25. Nakano V, Ignacio A, Llanco L, Bueris V, Sircili MP, Avila-Campos MJ: **Multilocus sequence typing analyses of Clostridium perfringens type A strains harboring tpeL and netB genes**. *Anaerobe* 2017, **44**:99-105.

26. Yonogi S, Matsuda S, Kawai T, Yoda T, Harada T, Kumeda Y, Gotoh K, Hiyoshi H, Nakamura S, Kodama T *et al*: **BEC, a novel enterotoxin of Clostridium perfringens found in human clinical isolates from acute gastroenteritis outbreaks**. *Infection and immunity* 2014, **82**(6):2390-2399.

27. Yonogi S, Kanki M, Ohnishi T, Shiono M, Iida T, Kumeda Y: **Development and application of a multiplex PCR assay for detection of the Clostridium perfringens enterotoxin-encoding genes cpe and becAB**. *J Microbiol Methods* 2016, **127**:172-175.

28. Rossjohn J, Feil SC, McKinstry WJ, Tweten RK, Parker MW: **Structure of a cholesterol-binding, thiol-activated cytolysin and a model of its membrane form**. *Cell* 1997, **89**(5):685-692.

29. Awad MM, Ellemor DM, Boyd RL, Emmins JJ, Rood JI: **Synergistic effects of alpha-toxin and perfringolysin O in Clostridium perfringens-mediated gas gangrene**. *Infection and immunity* 2001, **69**(12):7904-7910.

30. Moe PC, Heuck AP: **Phospholipid Hydrolysis Caused by Clostridium perfringens α-Toxin Facilitates the Targeting of Perfringolysin O to Membrane Bilayers**. *Biochemistry* 2010, **49**(44):9498-9507.

31. Deguchi A, Miyamoto K, Kuwahara T, Miki Y, Kaneko I, Li J, McClane BA, Akimoto S: **Genetic Characterization of Type A Enterotoxigenic Clostridium perfringens Strains**. *PLOS ONE* 2009, **4**(5):e5598.

32. Matsushita O, Yoshihara K, Katayama S, Minami J, Okabe A: **Purification and characterization of Clostridium perfringens 120-kilodalton collagenase and nucleotide sequence of the corresponding gene**. *Journal of bacteriology* 1994, **176**(1):149-156.
