# Supplementary material for: Dynamics of toxigenic Clostridium perfringens colonisation in a cohort of prematurely born neonatal infants
Source: BMC Pediatr. 2020 Feb 18;20:75. doi: 10.1186/s12887-020-1976-7 (PMC7027286; doi:10.1186/s12887-020-1976-7)
Supplement: Supplementary file 2 — Additional file 2. PCR methods for toxin gene detection. [file 12887_2020_1976_MOESM2_ESM.docx]

## Supplementary Material 2

## Multiplex PCR

The multiplex PCR protocol of van Asten *et al*. [1] was used to screen for the presence of *cpa*, *cpb*, *iA*, *etx*, *cpe* and *cpb2*. Each 50 μl reaction contained 1 μl DNA template (prepared by suspending colonies in distilled water, heating at 95 °C for 10 minutes and centrifuging at 17,000 xg^-1^), 7.5 μl 10 nM primer mix (Table 2), 10 μl 5x Taq Master Mix (New England Biolabs), 31.5 μl ultra-pure water. Thermal cycling consisted of initial denaturation at 94 °C for 1 minute followed by 34 cycles of denaturation at 94 °C for 20 seconds, annealing at 53 °C for 1 minute, extension at 68 °C for 1 minute, followed by final extension at 68 °C for 1 minute.

Table 2. *C. perfringens* toxin gene multiplex PCR primers.

| Primer | Gene | Sequence (5’-3’) | Product length (bp) |
| --- | --- | --- | --- |
| AlphaF | *cpa* | GCTAATGTTACTGCCGTTGA | 324 |
| AlphaR |  | CCTCTGATACATCGTGTAAG |  |
| BetaF | *cpb* | GCGAATATGCTGAATCATCTA | 195 |
| BetaR |  | GCAGGAACATTAGTATATCTTC |  |
| Beta2F | *cpb2* | AGATTTTAAATATGATCCTAAC | 548 |
| Beta2R |  | CCAATACCCTTCACCAAATACTC |  |
| EnterotoxinF | *cpe* | GGAGATGGTTGGATATTAGG | 485 |
| EnterotoxinR |  | GGACCAGCAGTTGTAGATA |  |
| EpsilonF | *etx* | GCGGTGATATCCATCTATTC | 376 |
| EpsilonR |  | CCACTTACTTGTCCTACTAAC |  |
| IotaF | *iA* | ACTACTCTCAGACAAGACAG | 272 |
| IotaR |  | CTTTCCTTCTATTACTATACG |  |

Primer pairs used in the multiplex PCR to target six toxin genes, the oligonucleotide sequence of the primers, and expected product size [1]*.*

## Duplex PCR

A duplex PCR was carried out to screen for BEC genes *becA* and *becB* as in Yonogi *et al* [2]. Each 25 μl reaction contained 1 μl DNA template, 0.5 μl of each primer (Table 3), 5 μl 5x Taq Master Mix (New England Biolabs) and 17 μl ultra-pure water. Thermal cycling consisted of initial denaturation at 94 °C for 3 minutes followed by 34 cycles of denaturation at 94 °C for 30 seconds, annealing at 53 °C for 30 seconds, extension at 68 °C for 1 minute, followed by final extension at 68 °C for 5 minutes.

**Table 3. BEC gene duplex primers.**

| Primer | Gene | Sequence (5’-3’) | Product length (bp) |
| --- | --- | --- | --- |
| becA F | *becA* | caatggggcgaagaaaatta | 324 |
| becA R |  | aaccatgatcaattaaaacctca |  |
| becB F | *becB* | tgcaaatgacccttacactga | 195 |
| becB R |  | agattggagcagagccagaa |  |

Primer pairs used in the duplex PCR to target becA/B toxin genes, the oligonucleotide sequence of the primers, and expected product size [2]

# Single PCR

Single PCRs were carried out for *netB, pfoA*, and *colA* genes using the primers shown in table 4. The following PCR reaction mix was used: 0.5 µl of each primer (Table 4), 5 µl 5x Taq Master Mix (New England Biolabs, UK), 1 µl template DNA and 18 µl of ultra-pure water. Thermal cycling settings for *netB* consisted of initial denaturation at 94 °C for 3 minutes followed by 34 cycles of denaturation at 94 °C for 30 seconds, annealing at 55 °C for 30 seconds, extension at 68 °C for 1 minute, followed by final extension at 68 °C for 5 minutes. Thermal cycling settings for *pfoA* and *colA* consisted of initial denaturation at 94 °C for 2 minutes followed by 34 cycles of denaturation at 94 °C for 30 seconds, annealing at 55 °C for 1 minute, extension at 68 °C for 1 minute, followed by final extension at 68 °C for 8 minutes.

**Table 4. Single PCR primers for *netB*, *pfoA* and *colA*.**

| Primer | Gene | Sequence (5’-3’) | Product length (bp) |
| --- | --- | --- | --- |
| netB F | *netB* | CGCTTCACATAAAGGTTGGAAGGC | 316 |
| netB R |  | TCCAGCACCAGCAGTTTTTCCT |  |
| pfoA F | *pfoA* | CAAGTATTGCAATGGCTTTATGTCTG | 866 |
| pfoA R |  | CTTTATAAGAGCTTTGAAAGCAGCTTG |  |
| colA F | *colA* | ATTAGAAAGTTTATGTACAATAGGTG | 816 |
| colA R |  | AAGACATTCTATTATTTCTATCGTAAGC |  |

Primer pairs used in individual PCRs to target netB [3], pfoA [4] and colA [4] toxin genes, the oligonucleotide sequence of the primers, and expected product size.

**PCR Controls**

Positive and negative PCR controls were run using typed strains with known presence of toxin genes. For the *cpa*, *pfoA* and *colA* genes the ATCC 13124 strain was used and for the *cpb*, *etx* and *cpb2* genes the ATCC 3626 strain was used. For the other genes ATCC controls were sought but were not available, hence strains sequenced within the lab and known to contain the appropriate toxin genes were used.

**Gel electrophoresis**

Multiplex PCR products were visualised on a 2.5% agarose gel, and all other PCR products visualised on 1.5% agarose gels stained with ethidium bromide, using 10 µl of reaction mix and 2 µl of 5x blue loading buffer. The loading mix was run at 125 V, 400 mA for 60 minutes alongside a 50 bp ladder and the gel visualised using a Chemidoc Gel Imager (Bio-Rad) and analyzed by Image Lab software (Bio-Rad).

**References**

1. van Asten AJAM, van der Wiel CW, Nikolaou G, Houwers DJ, Gröne A: **A multiplex PCR for toxin typing of Clostridium perfringens isolates**. *Veterinary Microbiology* 2009, **136**(3):411-412.

2. Yonogi S, Matsuda S, Kawai T, Yoda T, Harada T, Kumeda Y, Gotoh K, Hiyoshi H, Nakamura S, Kodama T *et al*: **BEC, a novel enterotoxin of Clostridium perfringens found in human clinical isolates from acute gastroenteritis outbreaks**. *Infection and immunity* 2014, **82**(6):2390-2399.

3. Keyburn AL, Boyce JD, Vaz P, Bannam TL, Ford ME, Parker D, Di Rubbo A, Rood JI, Moore RJ: **NetB, a New Toxin That Is Associated with Avian Necrotic Enteritis Caused by Clostridium perfringens**. *PLoS Pathogens* 2008, **4**(2):e26.

4. Deguchi A, Miyamoto K, Kuwahara T, Miki Y, Kaneko I, Li J, McClane BA, Akimoto S: **Genetic Characterization of Type A Enterotoxigenic Clostridium perfringens Strains**. *PLOS ONE* 2009, **4**(5):e5598.
